# Supplementary material for: Sustained-input switches for transcription factors and microRNAs are central building blocks of eukaryotic gene circuits
Source: Genome Biol. 2013 Aug 23;14(8):R85. doi: 10.1186/gb-2013-14-8-r85 (PMC4054853; doi:10.1186/gb-2013-14-8-r85)
Supplement: Additional file 5 — HTML Browsable Motif Output. Zipped folder containing all WaRSwap and FANMOD motif output, viewable in a web browser. [file gb-2013-14-8-r85-S5.ZIP › HTML_browsable_motif_output/WaRSwap_ath_tair9/sigs_WSWRSLDraw_2-2000.pvals.heatmaps.html/motif_id_46_000101110_tftype_ath_upstream_-1000_0.html]

```
BG_MODEL = Rnew2
MOTIF_ID = 46_000101110
TF_TYPE = ath
UPSTREAM = -1000_0


PVals
FN_0.2	FN_0.4	FN_0.6	FN_0.8
dg_60.genes	0.028	0	0.293	0.112
dg_70.genes	0.029	0	0.281	0.112
dg_80.genes	0.034	0	0.295	0.099

ZScores
FN_0.2	FN_0.4	FN_0.6	FN_0.8
dg_60.genes	1.7	2.805	0.365	-0.329
dg_70.genes	1.705	2.878	0.368	-0.324
dg_80.genes	1.724	2.915	0.346	-0.308

StDevs
FN_0.2	FN_0.4	FN_0.6	FN_0.8
dg_60.genes	106.615	42.39	6.333	0.431
dg_70.genes	104.944	41.561	6.446	0.438
dg_80.genes	103.994	40.273	6.444	0.386
```
